# Andexanet alfa for reversal of intracerebral haemorrhage resulting in ST-segment myocardial infarction: a case report

**DOI:** 10.1093/ehjcr/ytaf136

**Published:** 2025-03-24

**Authors:** Shaheen Mehrara, Ravi Patel, Nathan DeRon

**Affiliations:** Department of Internal Medicine, Methodist Dallas Medical Center, 1441 North Beckley Avenue, Dallas, TX 75203, USA; Department of Internal Medicine, Methodist Dallas Medical Center, 1441 North Beckley Avenue, Dallas, TX 75203, USA; Department of Internal Medicine, Methodist Dallas Medical Center, 1441 North Beckley Avenue, Dallas, TX 75203, USA

**Keywords:** Andexanet alfa, Myocardial infarction, Thromboembolism, Bleeding, Atrial fibrillation, Case report

## Abstract

**Background:**

Direct oral anticoagulants, such as direct factor Xa inhibitors, are commonly used to treat and prevent blood clots in patients with atrial fibrillation. Andexanet alfa reverses direct factor Xa inhibitors to help treat various thromboembolic conditions. Although it efficiently reverses anticoagulation by preventing life-threatening bleeding events, the use of andexanet alfa in today’s clinical practice is limited due to the risk of thromboembolism and cost.

**Case summary:**

A 76-year-old male presented with atrial fibrillation with rapid ventricular response and was started on Eliquis after a successful direct current cardioversion. Subsequently, the patient developed intracerebral haemorrhage, which required the administration of andexanet alfa. Ultimately, the patient suffered an ST-segment elevation myocardial infarction from complete occlusion of the mid-left anterior descending artery. His hospital course was complicated by acute hypoxic respiratory failure, septic shock, and renal failure. The family elected for comfort care measures and the patient expired shortly after.

**Discussion:**

This case emphasizes the need for individualized clinical assessment and collaboration with an interdisciplinary team regarding the use of andexanet alfa, as well as strategies to ensure thrombotic risk is effectively minimized. Current European Society of Cardiology Guidelines do not have recommendations for the use of andexanet alfa for the reversal of anticoagulation due to its uncertain risk profile. This case emphasizes the increased risk of thrombotic complications associated with andexanet alfa and highlights the importance of the continued need to research its use, which may help elucidate or revise current guidelines.

**Learning points**

- When using andexanet alfa, healthcare providers must be aware of the rare but serious risk of ST-segment myocardial infarction, a life-threatening complication.
- A multidisciplinary team approach is crucial to ensure the proper and safe administration of andexanet alfa.
- When andexanet alfa is clinically warranted, healthcare providers should carefully consider the potential risks of thrombotic events against the anticipated benefits of reversing anticoagulation and exercise appropriate clinical judgment.
- Additional research comparing the efficacy and safety profile of andexanet alfa with other anticoagulation reversal agents is needed, which would enable clinicians to make more well-informed treatment decisions.

## Introduction

Direct oral anticoagulants (DOACs) include the direct thrombin inhibitor, dabigatran, and four direct factor Xa inhibitors. Current data suggests that DOACs are non-inferior to warfarin in most disease states and have improved benefits, including lower rates of intracerebral bleeding, fewer drug and dietary interactions, and no need for drug level monitoring.^1,2^ Although effective, the main complication with their use is bleeding, which occurs in up to 4% of patients per year.^3^ Despite the clinical efficacy of andexanet alfa in reversing the effects of direct factor Xa inhibitors, there are infrequent but possible risks of thrombotic events, such as myocardial infarction (MI), ischaemic stroke, and thromboembolism.^4^ Here, we present the case of a patient who developed an acute ST-segment MI (STEMI) following initiation of andexanet alfa in an attempt to minimize intracerebral haemorrhage after apixaban administration.

## Summary figure

**Table:** 

Hospital Day 1	Presentation with shortness of breath. Initial attempts at rate control with metoprolol and diltiazem were unsuccessful. Electrocardiogram (EKG) showed the patient in atrial fibrillation with rapid ventricular response (RVR) and peak heart rate of 143 b.p.m. Admitted to ICU for further evaluation.
Hospital Day 2	Transoesophageal echocardiogram completed, which ruled out left atrial thrombus. Underwent successful direct current cardioversion with conversion to normal sinus rhythm. Initiated on apixaban.
Hospital Day 3	Woke up with expressive aphasia. STAT computed tomography scan of the head showed a rounded haemorrhagic lesion in the left posterior frontal lobe. Multidisciplinary team decided to administer andexanet alfa with 800 mg bolus and a maintenance infusion at 8 mg/min. Later that day, he developed chest pain with troponin rise to 7723 ng/L. Electrocardiogram was normal compared with baseline.
Hospital Day 4	Chest pain continued the following morning with a troponin peak of 14 240 ng/L. Initiated on therapeutic heparin infusion after repeat EKG consistent with a non-ST-segment elevation myocardial infarction (NSTEMI).
Hospital Day 5	Repeat EKG obtained for worsening chest pain consistent with a STEMI. Taken for an emergent left heart catheterization which showed 100% occlusive thrombus involving the mid-left anterior descending artery requiring balloon angioplasty to avoid triple therapy. Given loading dose of Plavix and started on IV heparin. Plans for staged percutaneous coronary intervention later during hospitalization.
Hospital Day 7	Developed acute hypoxic respiratory failure secondary to left hemothorax and pericardial effusion. Chest tube placed by interventional radiology. Plavix and heparin held.
Hospital Day 8	Chest tube clotted-off resulting in worsening respiratory failure. Developed septic shock requiring norepinephrine infusion. Started on IV antibiotics after blood cultures grew methicillin-resistant *Staphylococcus epidermidis*.
Hospital Day 9	Anuria developed. Continuous renal replacement therapy initiated.
Hospital Day 12	Family elected for comfort care measures. Do not resuscitate order placed. Patient expired.

## Case presentation

A 76-year-old male with a past medical history of chronic lymphocytic leukaemia (CLL) on ibrutinib, hypertension, and stage IV chronic kidney disease presented to the emergency department with shortness of breath. On physical exam, he was tachycardic with jugular venous distention up to his mandible, 2+ pitting oedema in bilateral lower extremities and crackles in his lower lung fields. His initial haemoglobin was 8.8 g/dL, platelet count was 348 000 platelets/µL, creatinine was 2.5 mg/dL, and estimated glomerular filtration rate was 26 mL/min. On admission, the patient was in atrial fibrillation (AF) with rapid ventricular response and a peak heart rate of 143 b.p.m. Initial attempts at rate control with metoprolol tartrate and diltiazem were unsuccessful. Transoesophageal echocardiography (TEE) followed by direct current cardioversion (DCCV) was required to correct the patient’s rhythm. He converted to normal sinus rhythm and was initiated on apixaban. A post-cardioversion transthoracic echocardiogram showed a mildly reduced ejection fraction (40–50%), with no evidence of wall motion abnormalities, valvular issues, or thrombus formation in the left atrium.

**Figure 1:**
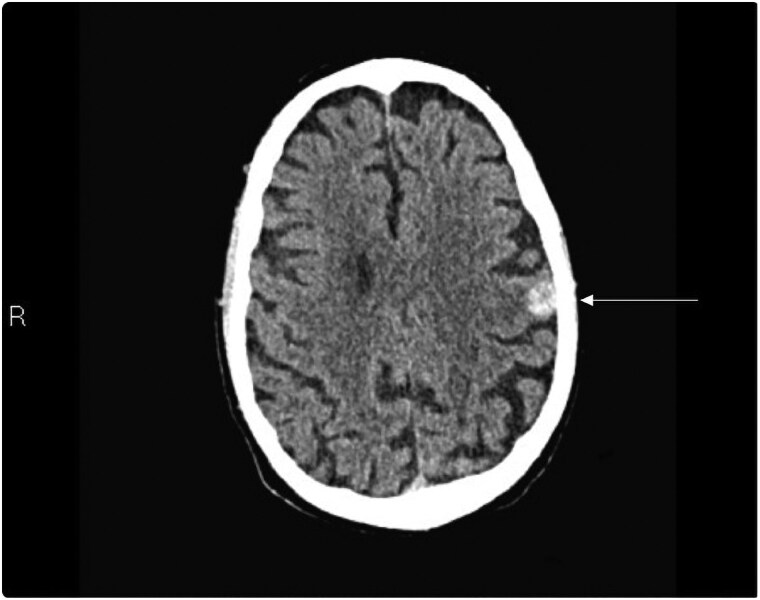
Non-contrast computed tomography scan axial images of the head demonstrating an acute haemorrhage in the left parietal lobe (white arrow).

**Figure 2:**
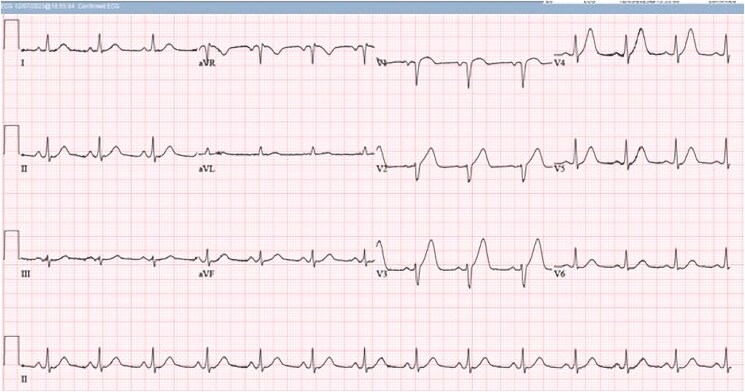
Baseline electrocardiogram of the patient following successful direct current cardioversion without any acute ischemic changes.

**Figure 3:**
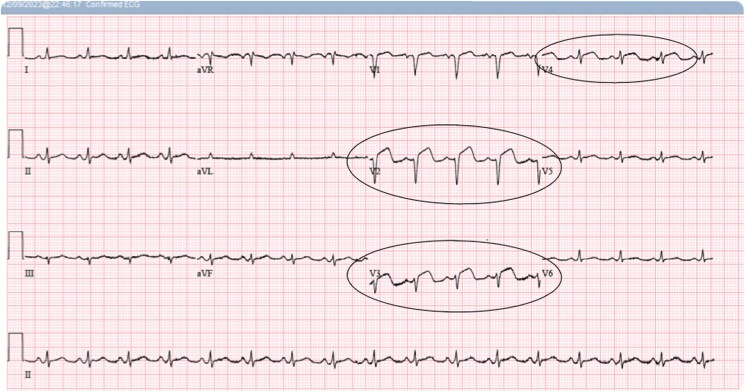
Electrocardiogram showing acute ST-segment elevation in anteroseptal leads V1, V2, V3 approximately 22 h after administration of andexanet alfa.

**Figure 4:**
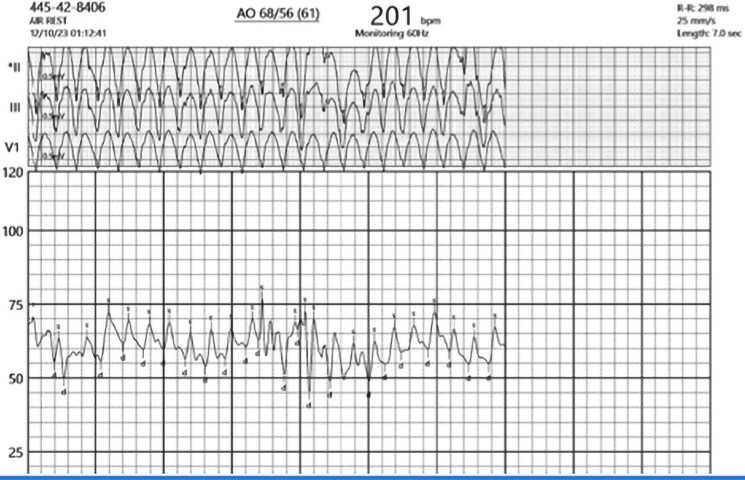
Telemetry monitor showing ventricular tachycardia at a rate of 201 b.p.m. during left heart catheterization for ST-segment elevation myocardial infarction.

On hospital Day 1, the patient developed expressive aphasia with computed tomography imaging notable for a rounded haemorrhagic lesion in the left posterior frontal lobe with a transverse diameter of 13 mm, which was concerning for brain haemorrhage (*Figure 1*). It was unclear if this was a primary haemorrhage or secondary to brain metastasis. Haematology/Oncology service was consulted and determined that the patient would benefit from andexanet alfa. He was bolused with 800 mg of andexanet alfa followed by a maintenance infusion at 8 mg/min. Later that day, the patient developed new-onset chest pain with high-sensitivity troponin-I rising from 207 ng/L (at presentation) to 7723 ng/L (normal range 0–20 ng/L. Creatinine Kinase-MB was not measured. However, a subsequent electrocardiogram (ECG) was grossly normal compared with the previous ECG without ischaemic changes (*Figure 2*). The following day, his troponin-I level peaked at 14 240 ng/L. He was initiated on a therapeutic heparin infusion after repeat ECG showed findings consistent with an NSTEMI. Approximately 22 h later, the patient’s chest pain worsened and a repeat ECG showed ST-segment elevation in the anteroseptal leads (*Figure 3*). He developed sustained pulseless ventricular tachycardia and advanced cardiovascular life support was initiated. He was administered an amiodarone bolus followed by continuous infusion and converted to sinus rhythm before reverting to AF with rapid ventricular response. A lidocaine infusion was given with return of spontaneous circulation. Emergent cardiac catheterization found a 100% mid-left anterior descending artery obstructive thrombus. Balloon angioplasty was employed without stent placement to avoid triple therapy with anticoagulation and antiplatelet medications. During the left heart catheterization, the patient required DCCV after developing multiple runs of ventricular tachycardia (*Figure 4*). The patient was given a loading dose of clopidogrel, and a heparin infusion was initiated with plans for staged percutaneous coronary intervention during hospitalization.

His clinical status deteriorated with the development of shock, possibly septic, after blood cultures speciated to methicillin-resistant *Staphylococcus epidermidis*. He developed worsening renal failure requiring continuous renal replacement therapy. The patient transitioned to comfort care and expired 9 days after andexanet alfa was administered.

## Discussion

Only two drugs have received Food and Drug Administration (FDA) approval for the reversal of DOACs: idarucizumab and andexanet alfa. Off-label use of haemostatic agents include activated prothrombin concentrate complex for dabigatran-associated bleeding and four-factor prothrombin complex concentrate for direct-Xa inhibitor-associated bleeding.^5^ Andexanet alfa binds the active site of factor Xa inhibitors and sequesters them, thus reducing anticoagulant effects while restoring the activity of endogenous factor Xa. This can be measured directly with anti-factor Xa level or indirectly by thrombin generation.^6^

The 2019 Anticoagulation Forum Guidelines recommend using reversal agents in the following cases only: life-threatening bleeding (as in our patient); bleeding into critical organs; urgent invasive procedures in DOAC-treated patients; other major bleeding not controlled with maximal support measures; or if there is reasonable suspicion that there is a clinically relevant plasma DOAC level.^5^ In a multicentre clinical study examining safety outcomes after andexanet alfa therapy, 34 (10%) patients on andexanet alfa experienced a thrombotic event within 30 days of initiation.^7^ Approximately one-third of these thrombotic events occurred within the first 5 days of initiation. This case highlights the different comorbidities and circumstances that contribute to complex clinical decision-making and the importance of collaborative care regarding the risk of adverse events with andexanet alfa use.

The FibStroke study was a multicentre cross-sectional study that evaluated stroke incidence in patients with AF. After cardioversion, 6.4% of strokes were in patients with paroxysmal/persistent AF. Most strokes and transient ischaemic attacks occurred shortly after successful cardioversion with a median time from cardioversion to event of 2 days. The risk of stroke after cardioversion guided by TEE is ∼0.8%.^8,9^ With the median time from cardioversion to stroke being 2 days, this patient’s time between cardioversion to stroke was atypical and reinforces that these agents should be used with caution and in conjunction with further specialists to ensure maximal patient safety.

There is a notable discrepancy in the cost between andexanet alfa and other anticoagulation reversal agents. Although effective in reducing life-threatening haemorrhage, the cost of andexanet alfa limits its use in clinical practice and further underscores the need to explore alternative options to reverse anticoagulation when needed.^10^

The patient’s concomitant diagnosis of CLL may have contributed to thromboembolism formation; however, his CLL was diagnosed ∼15 years prior and he was stable on maintenance immunotherapy. Ibrutinib use has been linked to pancytopenia and bleeding due to its effects on the bone marrow and coagulation cascade, but the patient’s platelet counts were normal. Ibrutinib use with DOACs has not been linked to excess bleeding, but more research is needed to help minimize the potential adverse effects.^11^ The TEE completed prior to the DCCV helped ensure there was no left atrial appendage thrombus, which decreased the likelihood of the DCCV leading to the STEMI. Andexanet alfa administration can induce a pro-coagulant state, causing temporary increases in D-dimer, prothrombin fragments 1 and 2, and endogenous thrombin potential.^12,13^ However, the clinical haemostatic efficacy of andexanet alfa does not appear to correlate with reductions in anti-Xa activity, making anti-Xa monitoring less useful for predicting clinical response. In patients with intracranial haemorrhage, anti-Xa reduction does predict haemostatic efficacy and is associated with lower mortality.^7,14^ The ANNEXA-1 trial further supported these findings.^15^ Monitoring anti-Xa activity in patients with MI or thrombotic events may help with risk stratification and managing complications. Monitoring D-dimer and prothrombin 1 and 2 levels may also be useful for anticipating and managing thrombotic complications. However, more research is needed to fully understand these laboratory parameters’ predictability, reliability, and efficacy in this context.

## Conclusion

This case underscores the delicate balance between anticoagulation reversal and thrombotic risk in critically ill patients. While andexanet alfa is an effective agent for reversing the anticoagulant effects of factor Xa inhibitors in life-threatening bleeding, its administration carries a significant risk of thrombotic complications, as demonstrated in our patient who suffered an acute STEMI. This case also highlights the different comorbidities and circumstances that contribute to complex clinical decision-making and the importance of collaborative care regarding the risk of adverse events with andexanet alfa use. Further research is needed to refine clinical guidelines, optimize patient selection, and identify strategies to mitigate thrombotic risks associated with andexanet alfa use.

## Data Availability

The data underlying this article will be shared on reasonable request to the corresponding author.

## Lead author biography

Shaheen Mehrara is a second-year internal medicine resident physician at Methodist Dallas Medical Center in Dallas, Texas. He is originally from Birmingham, Alabama, and received his medical degree from VCOM-Auburn in Auburn, Alabama. His interests include cardiovascular disease, cardiac pathology and electrophysiology.

## Notes

**Consent:** Complete written informed consent was obtained from the patient’s next of kin for the publication of this study.

**Funding:** The authors did not receive support from any organization for the submitted work.
